# Pest Alert Tool—a web-based application for flagging species of concern in metabarcoding datasets

**DOI:** 10.1093/nar/gkad364

**Published:** 2023-05-19

**Authors:** Anastasija Zaiko, Maximilian Scheel, Jessica Schattschneider, Ulla von Ammon, Michelle Scriver, Xavier Pochon, John K Pearman

**Affiliations:** Cawthron Institute, Private Bag 2, Nelson 7042, New Zealand; Institute of Marine Science, University of Auckland, Private Bag 92019, Auckland 1142, New Zealand; Sequench Ltd, 1/131 Hardy Street, Nelson 7010, New Zealand; Cawthron Institute, Private Bag 2, Nelson 7042, New Zealand; Cawthron Institute, Private Bag 2, Nelson 7042, New Zealand; Cawthron Institute, Private Bag 2, Nelson 7042, New Zealand; Cawthron Institute, Private Bag 2, Nelson 7042, New Zealand; Institute of Marine Science, University of Auckland, Private Bag 92019, Auckland 1142, New Zealand; Cawthron Institute, Private Bag 2, Nelson 7042, New Zealand; Institute of Marine Science, University of Auckland, Private Bag 92019, Auckland 1142, New Zealand; Cawthron Institute, Private Bag 2, Nelson 7042, New Zealand

## Abstract

Advances in high-throughput sequencing (HTS) technologies and their increasing affordability have fueled environmental DNA (eDNA) metabarcoding data generation from freshwater, marine and terrestrial ecosystems. Research institutions worldwide progressively employ HTS for biodiversity assessments, new species discovery and ecological trend monitoring. Moreover, even non-scientists can now collect an eDNA sample, send it to a specialized laboratory for analysis and receive in-depth biodiversity record from a sampling site. This offers unprecedented opportunities for biodiversity assessments across wide temporal and spatial scales. The large volume of data produced by metabarcoding also enables incidental detection of species of concern, including non-indigenous and pathogenic organisms. We introduce an online app—Pest Alert Tool—for screening nuclear small subunit 18S ribosomal RNA and mitochondrial cytochrome oxidase subunit I datasets for marine non-indigenous species as well as unwanted and notifiable marine organisms in New Zealand. The output can be filtered by minimum length of the query sequence and identity match. For putative matches, a phylogenetic tree can be generated through the National Center for Biotechnology Information’s BLAST Tree View tool, allowing for additional verification of the species of concern detection. The Pest Alert Tool is publicly available at https://pest-alert-tool-prod.azurewebsites.net/.

## Graphical Abstract

**Graphical Abstract:**
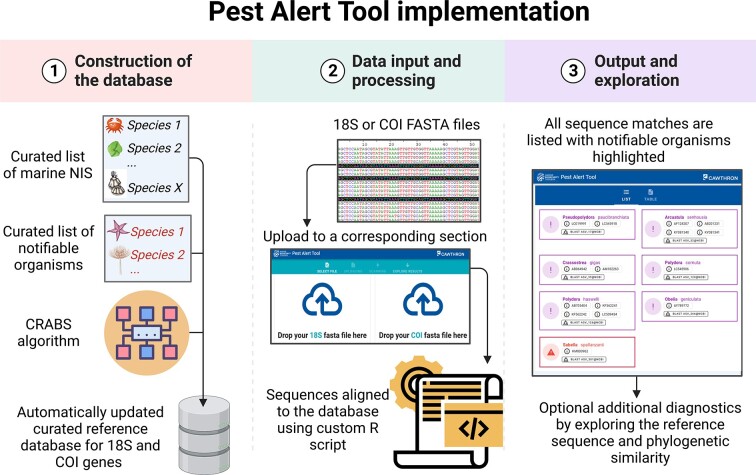
Implementation of the Pest Alert Tool and its functionality.

## INTRODUCTION

Rapid development of high-throughput sequencing (HTS) technologies has revolutionized our ability to characterize biodiversity across ecosystems. They fueled ubiquitous applications of environmental DNA (eDNA) metabarcoding—a genetic method that amplifies homologous gene(s) across species to derive taxonomic constituents of a community (1)—for new species discovery, ecological trend monitoring and environmental impact assessments (2–4). One of the increasingly touted eDNA metabarcoding applications is for biosecurity surveillance (5–7), a field specifically devoted to monitoring environments for pest species with potentially devastating ecological and economic consequences. The large volume of data produced by metabarcoding also raises the possibility of upscaling biosecurity surveillance capabilities, enabling indeliberate detection of species of concern through the broad assessment of entire biological communities (8, 9). The allure of this ‘passive surveillance’ approach is obvious: we do not always know which species of concern to expect, and casting a broad surveillance net across the taxonomic spectrum may allow early detection of new, unanticipated pest incursions. More broadly, passive surveillance could enable efficient leveraging of resources, as HTS technologies are increasingly applied for biodiversity monitoring purposes across a variety of biomes and contexts (10–13) and can be secondarily adopted for biosecurity surveillance (8).

Reporting unverified biosecurity risks from HTS data, however, may lead to a rapid increase in workload for biosecurity managers for minimal benefit, and can even lead to legal actions against researchers in case of false-positive reporting (8). Furthermore, researchers conducting HTS-based biodiversity surveys might be unaware of the biosecurity threats occurring in their region and often overlook ramifications with key end users concerned with the spread of unwanted organisms. Therefore, there is a growing interest in simple tools that could alert both researchers and stakeholders to the potential biosecurity risks contained in HTS datasets (14). For instance, straightforward web-based informatics tools that screen pre-publication HTS datasets for the presence of species of concern could be implemented as part of best practice standards in eDNA-based research and biomonitoring (15) and allow researchers to conduct important secondary quality assurance steps.

We introduce the Pest Alert Tool, an online server (application), developed for screening HTS datasets for species of concern, and showcase its applicability for secondary quality assurance steps or reporting of putative threats to environmental agencies. This is an open tool, which combines an algorithm for the automatic generation and update of a reference database based on a curated list of species of interest and BLAST processing of submitted FASTA files in a user-friendly interface. The current version of the tool was created as a proof of concept to address the immediate demand of New Zealand biosecurity practitioners and is set up to screen nuclear small subunit 18S ribosomal RNA (18S rRNA) and mitochondrial cytochrome oxidase subunit I (COI) genes for marine non-indigenous species (NIS) as well as unwanted and notifiable marine organisms in New Zealand. However, it can easily be adjusted for other regions or applications following the guidelines provided in this account.

## MATERIALS AND METHODS

### Implementation

**Figure 1.**
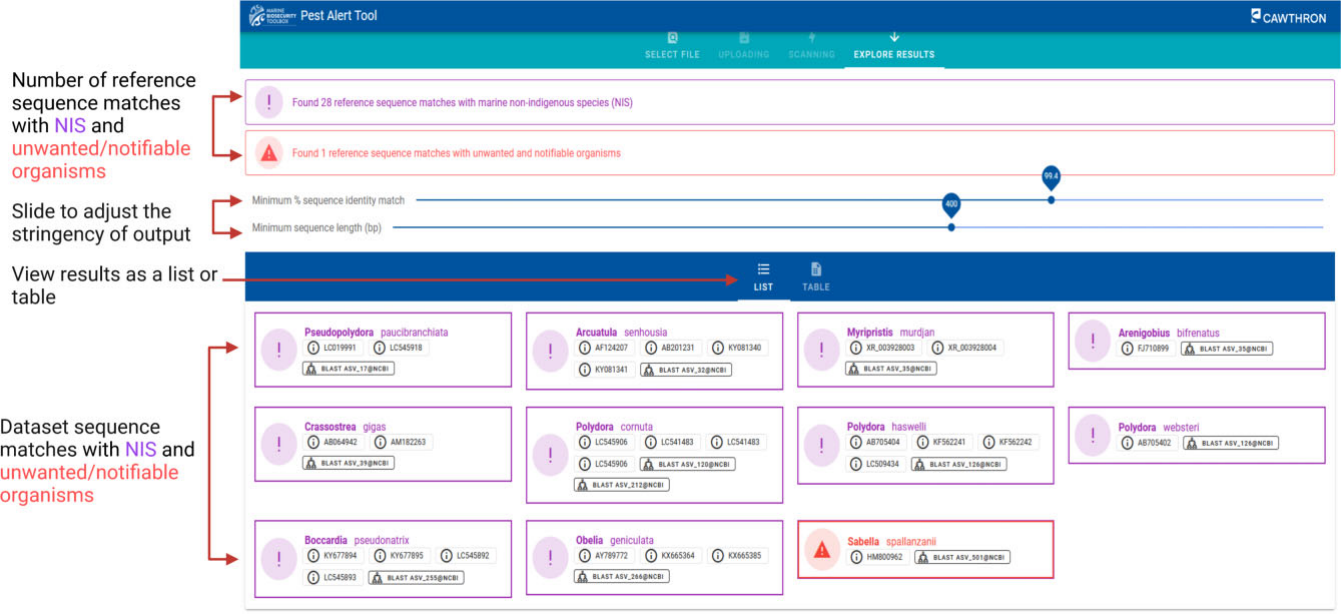
Layout of the results generated by the Pest Alert Tool.

The Pest Alert Tool is based on comparing HTS files in FASTA format against automatically generated customized nuclear 18S rRNA and COI gene databases (Figure 1). These databases consist of all the sequences belonging to genera that contain species that have been identified as marine pests in New Zealand, i.e. NIS (https://pest-alert-tool-prod.azurewebsites.net/examples/NIS_list_22-11-17.txt) and species on Biosecurity New Zealand’s list of unwanted and notifiable marine organisms (https://pest-alert-tool-prod.azurewebsites.net/examples/Unwanted_notifiable_22-11-17.txt). The databases were constructed using the Creating Reference databases for Amplicon-Based Sequencing (CRABS) algorithm (16) with sequences obtained from National Center for Biotechnology Information (NCBI) (17) for 18S rRNA and COI and Barcode of Life Data System (18) for COI only. The integrated CRABS algorithm allowed automated construction of the databases and updates every 6 months. Sequences acquired by CRABS are quality checked (minimum length 250 bp; maximum number of N’s = 1) and result in two clean FASTA files (one for 18S rRNA and one for COI). The FASTA files are then converted into databases, using the makeblastdb function, which can be used to align the submitted sequences against. The user can stay informed on the latest updates by checking the disclaimer box at the bottom of the page, which also provides information on reference database coverage for New Zealand marine NIS and unwanted and notifiable organisms. The instructions on how to use the tool can be accessed via the ‘HELP’ button in the upper panel (Supplementary File S1).

The minimum input required for the Pest Alert Tool is a text file containing DNA sequences originating from either the 18S rRNA or COI gene in a FASTA format (*.fa or *.fasta extension). FASTA files are text files, where each sequence begins with a single-line description, followed by a line of sequence data. The single-line description contains a greater than (>) symbol followed by the sequence name. These files can be opened and edited in any text-editing or word-processing program. Various length example FASTA files randomly compiled from published New Zealand marine datasets are downloadable on the Pest Alert Tool web page (e.g. https://pest-alert-tool-prod.azurewebsites.net/examples/100.fa). The input FASTA files are HTS output files pre-processed (i.e. filtered, trimmed, merged and denoised) with a bioinformatics pipeline [e.g. DADA2 (19)] prior to being uploaded into the Pest Alert Tool. Basic raw data processing is needed to reduce the screening time and potential error rate in the output results. Inexperienced HTS users commonly outsource the raw data processing steps to a sequencing or bioinformatics service provider and FASTA is the usual output file format they receive. For users with some experience in data processing, we provide a fully annotated guide on how to construct a FASTA file from the raw sequencing files following the DADA2 pipeline in R (Supplementary File S2).

Using the input FASTA file, the Pest Alert Tool queries the appropriate BLAST database using blastn (20) with the megablast algorithm. The BLAST is run with a maximum number of reported sequence matches set at 100.

To ensure consistency and ease of deployment, all backend services were containerized and managed through a single Docker Compose file. The API was constructed with FastAPI, a modern Python framework, and deployed using Gunicorn (https://gunicorn.org/) and NGINX (https://www.nginx.com/) web servers. The NGINX service also served the static content and ensured the availability and scalability of the website. To offload work from the application, we employed the Celery protocol (21). To improve performance, we utilized Redis (https://redis.com/) as a caching engine, providing an in-memory data structure store. On the front end, we used Vue.js (https://vuejs.org/), a progressive JavaScript framework for building user interfaces. This framework offers a component-based programming model based on standard HTML, CSS and JavaScript. The whole code base developed to build the Pest Alert Tool is available in a public Git repository (https://gitlab.com/cawthron-public/marine-biosecurity-toolbox/pest-alert-tool).

### Output

Once the FASTA file has been uploaded, the screening for putative pests commences instantly. When the screening is complete, the Pest Alert Tool will navigate to the ‘EXPLORE RESULTS’ screen (Figure 1). Here, users can see whether any of their submitted sequences match with New Zealand marine NIS or with unwanted and notifiable organisms. At the top, below the main toolbar, the number of reference sequence matches to marine NIS and notifiable organisms is reported. Values for the minimum percentage sequence identity match and minimum sequence length can be adjusted by the user to allow for variations in the stringency of the results. Allowed adjustment ranges are 98–100% for the minimum percentage sequence identity match and 100–600 for the minimum sequence length. By selecting lower percentage sequence identity values (i.e. <99%) and too short minimum sequence length threshold (i.e. <250 bp), the user decreases the specificity of the match, potentially increasing the risk of incorrect species identification. Therefore, additional verification steps for such matches are recommended.

Further verification of query sequences that have resulted in hits to NIS or notifiable species can be undertaken by investigating the reference sequences by pressing the i-button under each species name in a list view. A phylogenetic tree can also be created via pairwise alignments in NCBI (see below). As a rule of thumb, matches supported by multiple reference sequences are expected to be more robust. However, due to existing gaps in sequence reference databases, some species might have only one or few reference sequences available. It is advised that matches to putative pests are further inspected to verify the robustness of the reference, for example, by verifying whether additional information is provided on species ID, provenance and/or vouchered specimen.

For additional diagnostics of the sequence match specificity, it is highly recommended to verify the match by checking the phylogenetic tree of the wider range of related taxa from NCBI. This can be done directly from the Pest Alert Tool results in a list view mode, by pressing the ‘Tree BLAST’ button next to the species of interest. By pressing the button, the sequence from the user’s dataset is submitted to NCBI’s phylogenetic tree generation engine that runs BLAST pairwise alignments. When the tree is ready, a green button ‘VIEW TREE’ appears. This link on the button will take the user to the Tree View on NBCI platform. For more details about interacting with the Tree View on NCBI look at the NCBI tutorial (https://www.ncbi.nlm.nih.gov/tools/gbench/tutorial19/). To verify the match, users should check the location of the yellow highlighted query (the submitted sequence). A robust match is expected to be on a branch with the same named species and to form a separated phylogenetic clade from other species.

### Examples of use

The anticipated primary use of the Pest Alert Tool is screening of metabarcoding datasets generated from New Zealand marine samples (from routing monitoring, environmental assessments or any research-focused projects) to identify potential presence of the risky taxa. The recent outburst of HTS-based biodiversity data (22–24) often leads to the publication of taxonomic inventories that include species of concern without cautioning prospective end users on the potential for misassignment or other sources of false-positive errors (8). For example, there have been cases where identification of species of concern in already published HTS data raised management responses that led to additional quality control analyses demonstrating the erroneous attribution of these species (25, 26). Such responses may incur significant financial burden to governmental agencies and/or expose researchers to legal entanglements. Therefore, the Pest Alert Tool offers a simple solution for marine eDNA practitioners and environmental managers alike, enabling the rapid screening of sequence data pre- and post-publication, and hence represents a powerful verification platform for improved quality assurance standards of HTS data. We see the tool being adopted by metabarcoding data holders (researchers, environmental consultants, biosecurity practitioners, community groups, etc.) as a routine quality assurance step. This would allow informed decision on whether data owner needs to inform authorities on potential presence of species of concern, conduct additional verification steps or add a disclaimer on uncertainties associated with the detection and identification of these species (8). The tool can also be used retrospectively, on datasets already existing in the public domains, for example, to establish or verify the baselines of NIS incursions and distribution in the coastal waters. This would provide useful information for biosecurity managers and decision-makers on likely vectors of spread, population dynamics and potential future introductions to keep an eye on.

We also see potential applicability of the tool for research purposes. For example, as part of the initial proof-of-concept test, the offline algorithm underlying the Pest Alert Tool has been tested in a study aimed at investigating potential affinity of marine NIS to particular biopolymer types in a marine deployment experiment (27). The algorithm allowed to quickly screen an extensive dataset obtained from 60 biofilm samples, representing 4225 unique amplicon sequence variants (ASVs) for a subset of marine NIS ASVs. Now, with the algorithm implemented in the web-based server with a user-friendly interface, subsetting of metabarcoding datasets can easily be performed by researchers or students interested in marine pests independently of their proficiency in bioinformatics pipelines. The tool can also be offered as an educational resource for community outreach activities or science curriculum at high school to raise public awareness of marine environmental issues and novel biodiversity surveillance approaches offered by HTS tools.

### Future development

The Pest Alert Tool is intended initially to screen metabarcoding datasets for marine pests in New Zealand, but it can easily be adapted to detect other species of interest—freshwater or terrestrial species of concern, pathogens, and protected or indicator taxa. It can also be extended to other regions and other metabarcoding markers. The minimum requirement for such an extension is a curated list of species of interest. In Supplementary File S3, we provide detailed guidelines for how the tool can be adjusted to create a customized database for other regions or applications. The efficiency of the tool is reliant on the availability of reference sequences of target taxa; therefore, collaborative cross-national effort augmenting existing open access databases with quality references is warranted and of high priority for future developments. Moreover, with future improved knowledge of regional biodiversity and completeness of reference sequence databases, an extension to the screening server can be implemented to indicate indigenous biodiversity found in a sample and highlight known unwanted organisms, but also species that are not yet listed as unwanted or non-indigenous but are new to the region. A further extension to allow registering and tracking unwanted or suspicious detections in a searchable GIS format can be a useful live resource for informing targeted and precautionary management response to emerging biosecurity risks by governmental agencies.

### Citing the Pest Alert Tool

Authors who make use of the Pest Alert Tool web server should cite this article as a general reference and should also include https://pest-alert-tool-prod.azurewebsites.net/. The web server pages will list additional articles for citation that relate to the algorithms employed, the software that implements them and the energy parameters it uses.

## DATA AVAILABILITY

Pest Alert Tool is an open source collaborative initiative available in the GitLab repository (https://gitlab.com/cawthron-public/marine-biosecurity-toolbox/pest-alert-tool).

## Supplementary Material

gkad364_Supplemental_Files
