# Supplementary material for: Pest Alert Tool—a web-based application for flagging species of concern in metabarcoding datasets
Source: Nucleic Acids Res. 2023 May 19;51(W1):W438–42. doi: 10.1093/nar/gkad364 (PMC10320087; doi:10.1093/nar/gkad364)
Supplement: gkad364_Supplemental_Files [file gkad364_supplemental_files.zip › Supp1_Zaiko_etal_PAT_user_guide.docx]

# Supplementary 1: User Guide for Pest Alert Tool for Users

**Access Pest Alert Tool**


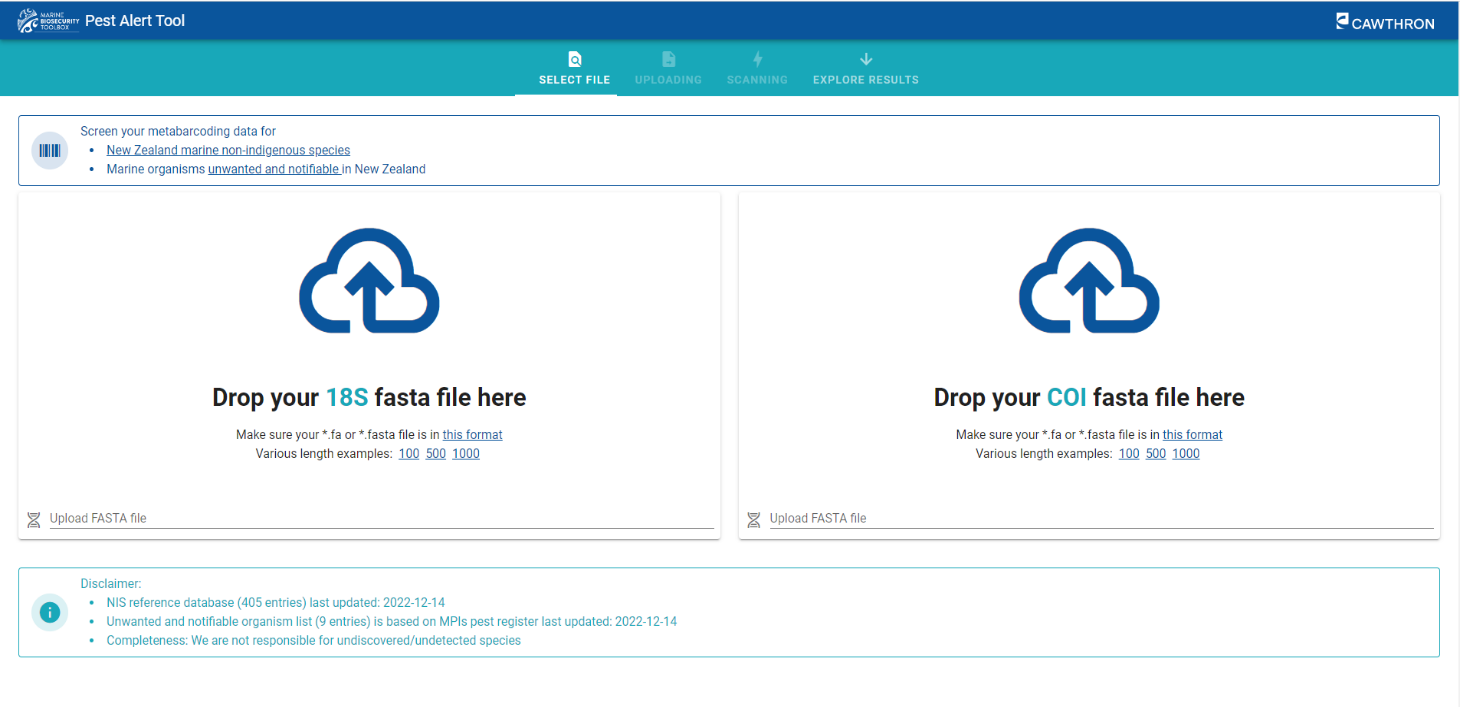
Users enter the homepage of the website at <https://pest-alert-tool-prod.azurewebsites.net/>

**Figure 1**: Homepage of Pest Alert Tool


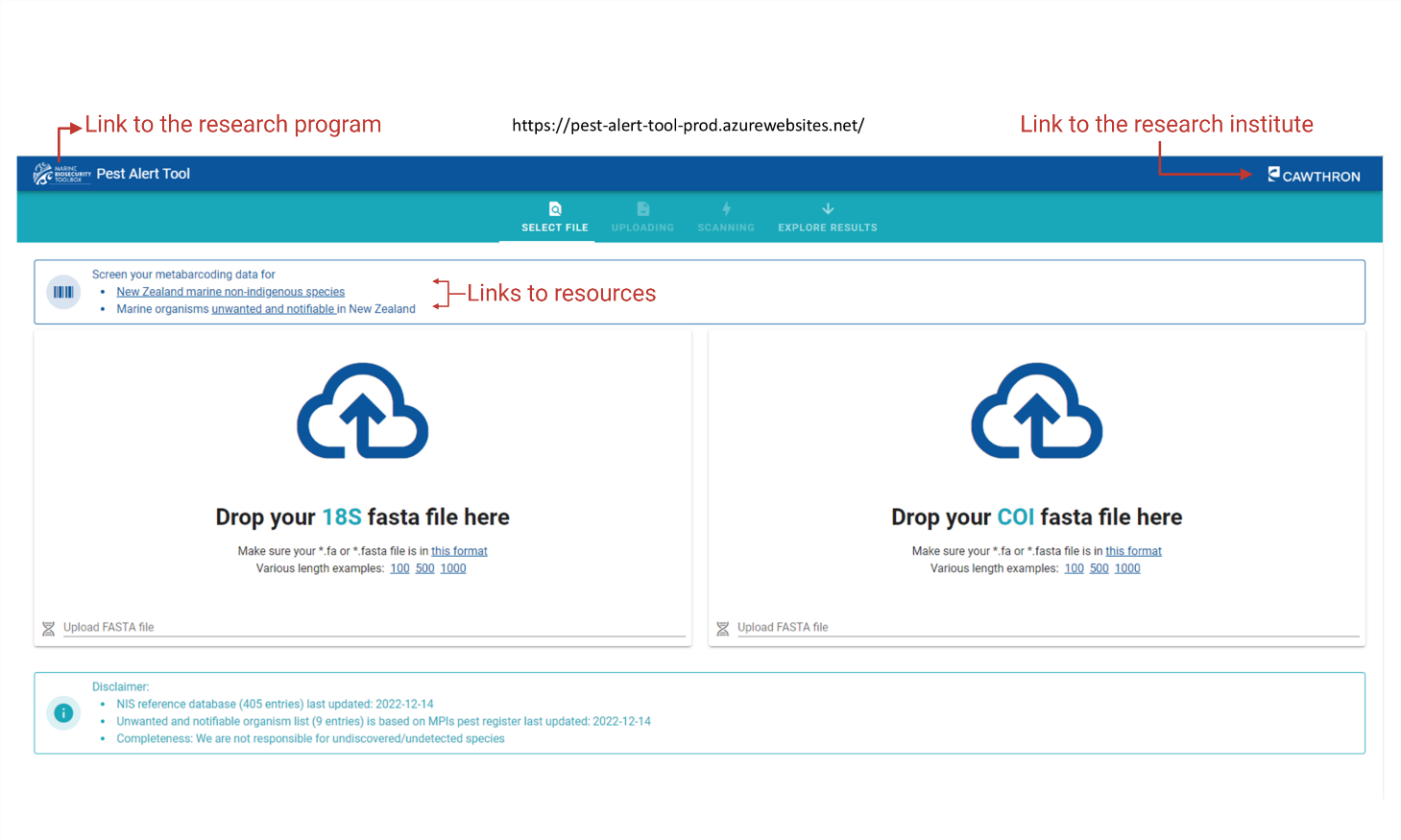
**Links to important resources**

**Figure 2:** Homepage of Pest Alert Tool with highlights on important resources.

On the homepage, users will find links to resources such as how metabarcoding data can be used for detecting marine pests: non-indigenous species (NIS) and Biosecurity New Zealand’s list of unwanted and notifiable marine organisms. As well as links to the research institute, Cawthron Institute, and the research program, the Marine Biosecurity Toolbox involved in creating the App and the research behind this tool.

The Disclaimer section at the bottom of the page provides information on reference database coverage of New Zealand marine NIS and unwanted and notifiable organisms, as well as the latest update information.

**Download input files examples**


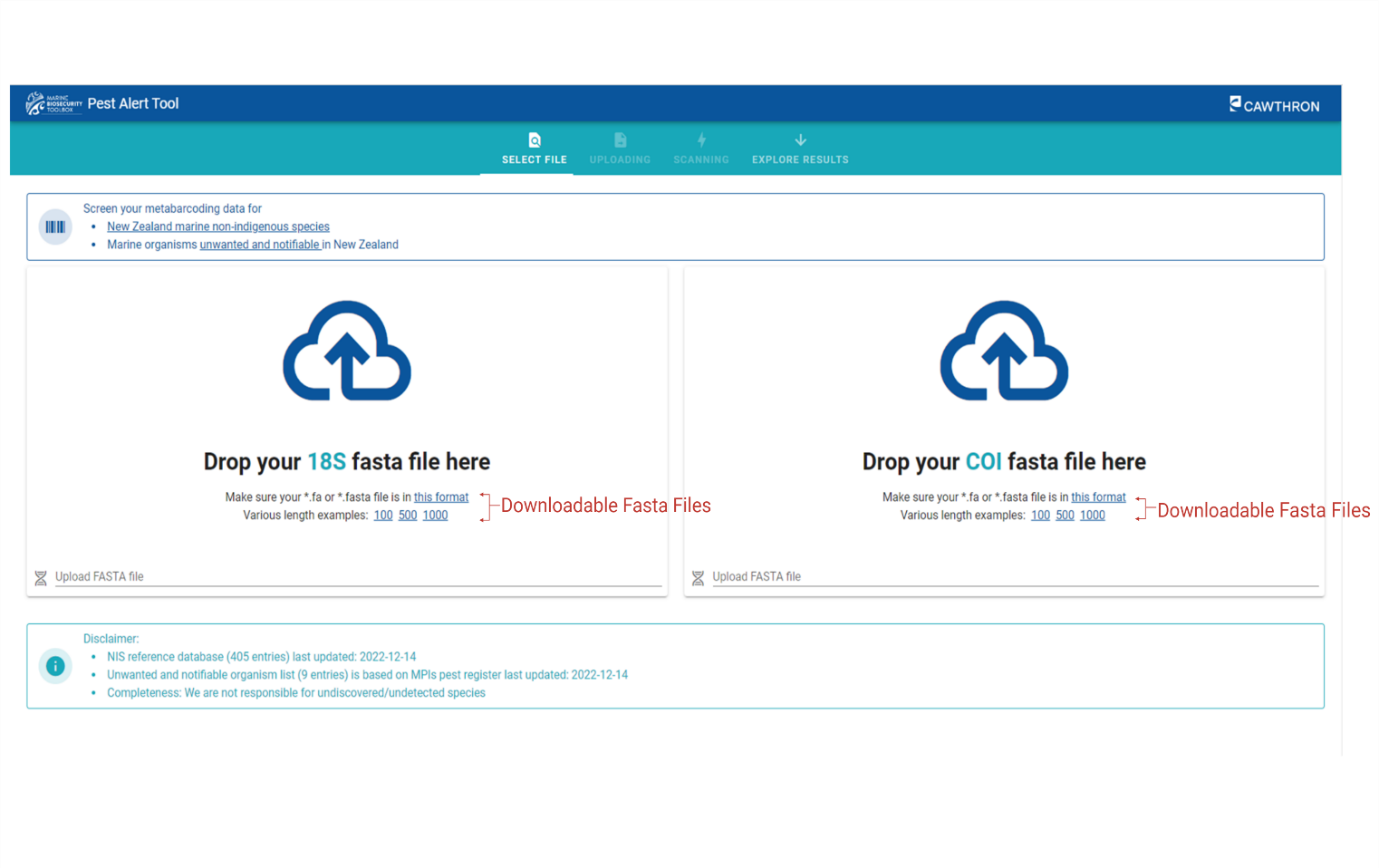
Prior to using this tool, users should ensure their input FASTA files (*fa or *fasta) are in the correct format. This format is known as FASTA files. FASTA files are text files, where each sequence begins with a single line description, followed by a line of sequence data. The single-line description contains a greater-than (>) symbol in the first column followed by the sequence name.

**Figure 3:** Homepage of Pest Alert Tool with highlights on where the download example FASTA files.

**
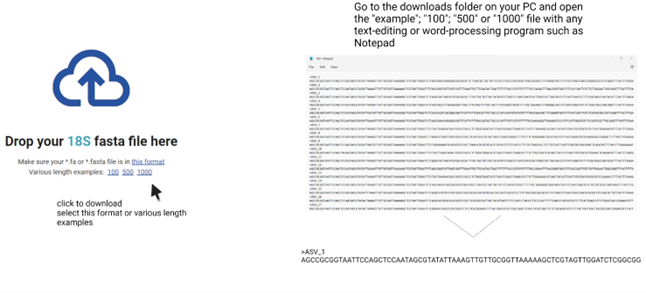
**

**Figure 4:** Example of what a FASTA file looks like once it is downloaded from the Pest Alert Tool homepage.

Examples of FASTA files are downloadable on the Pest Alert Tool, note these files are TXT files and can be opened and edited in any text-editing or word-processing program. The tool includes example FASTA files of various lengths (100,500,1000). These files can be submitted for a trial run.

**Inputting FASTA Files (sequencing data)**


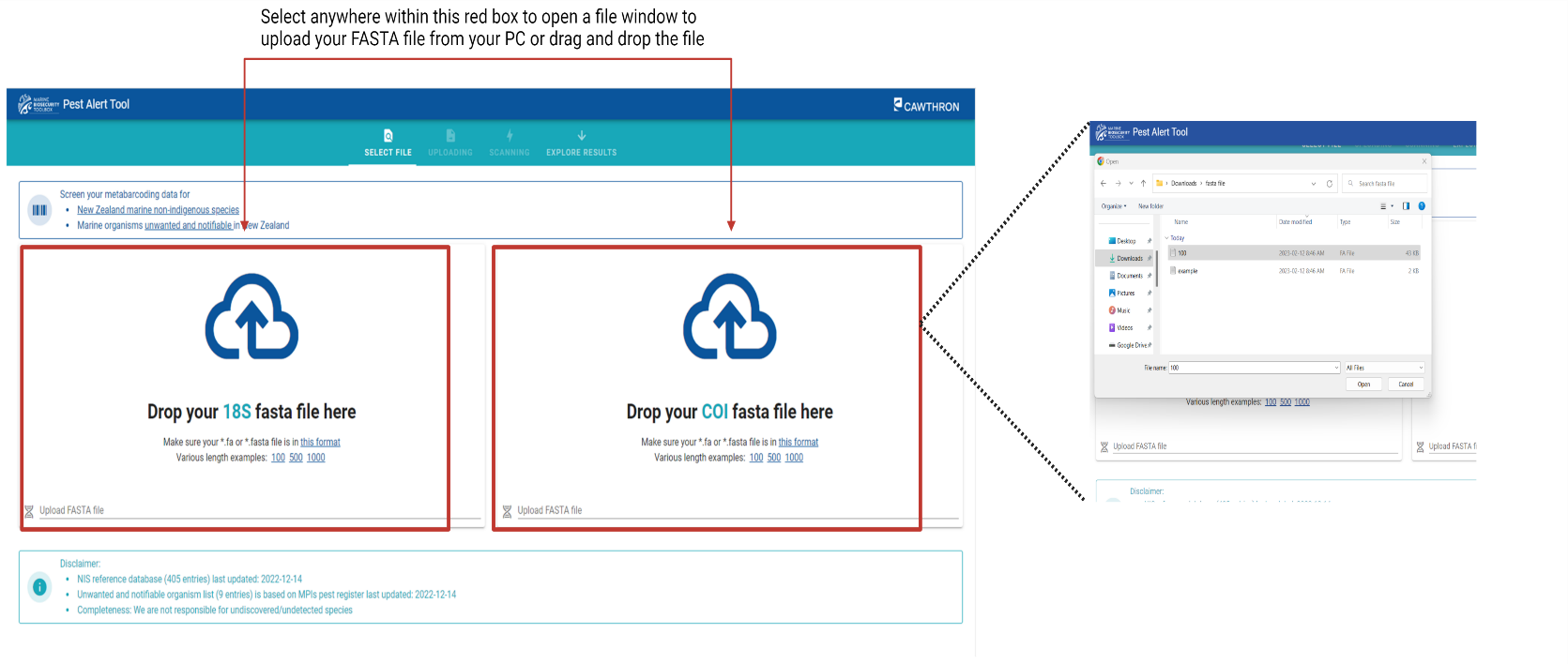
If the sequencing data is in the appropriate FASTA files (*fa or *fasta), see the section above, it can be inputted into the tool to screen for marine non-indigenous species as well as unwanted and notifiable marine organisms in New Zealand. To screen the Cytochrome c oxidase subunit I (COI) and nuclear small subunit 18S ribosomal RNA (18S rRNA) FASTA files, drag and drop the FASTA file into the appropriate location either “Drop your 18S fasta file here” or “Drop your COI fasta file”. Alternatively, FASTA files can be uploaded using the Browse – The file Upload Feature. Note this tool is only for sequence data that either targeted the 18S or COI region of the genome, see the link to resources for more details.

**Figure 5:** How to upload FASTA files on the Pest Alert Tool; either drag and drop the file within the red box or click anywhere in the red box to open the browse-file upload feature.

**Screening**


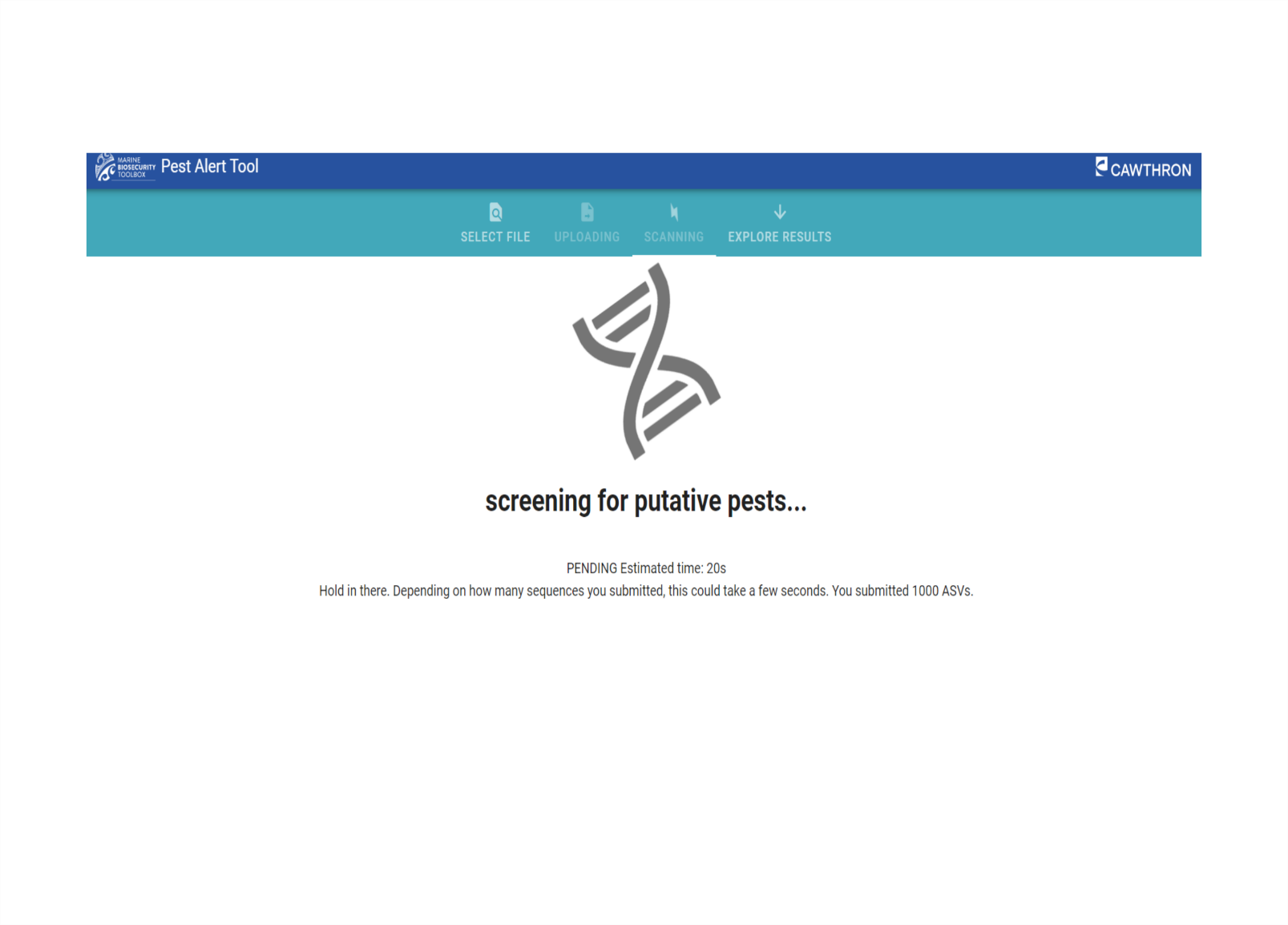
Once the FASTA file has been uploaded the screening for putative pests will commence. The tool will indicate an estimated time till the completion of screening, depending on the size of the dataset.

**Figure 6:** Pest Alert Tool scanning page, indicates the estimated time till screening for putative pests is complete.

**Explore Results Screen**

Once screening is complete, the Pest Alert Tool will navigate to the “explore results” screen. Here users can see if any of their submitted sequences match with New Zealand marine NIS or with unwanted and notifiable organisms. At the top below the main toolbar, a summary output will confirm how many reference sequences matches were found in your dataset. Users can adjust the stringency of the results by adjusting the minimum % sequence identity match (sequence similarity with the reference) and minimum sequence length (bp) sliders. The further the sliders are moved to the left, the less stringent the results and results are more stringent when the sliders are moved to the right. Users can adjust as needed based on the purpose of the study and dataset specifics. Once adjustments are made, users can explore the sequence matches in a list or table format. See the next section for details.


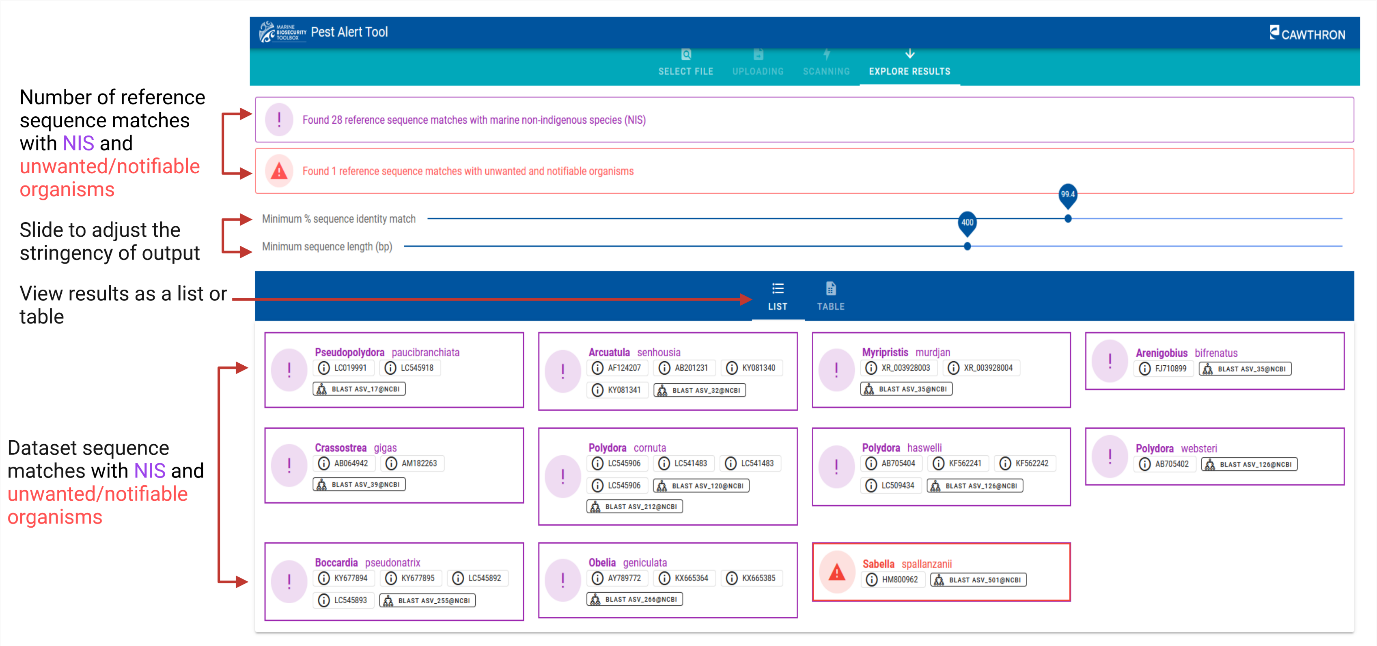
**Figure 7**: Explore Results, layout of the results generated by the Pest Alert Tool

**Additional diagnostics of the sequence matches**


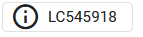
It is advised that matches to putative pests are further inspected by checking the reference sequences by pressing the (i) button under the species name in a list view, i.e. to verify the robustness of the reference, e.g.:

-where it comes from

-how much additional information provided on species ID and provenance

-does it come from a vouchered specimen, etc.

As a rule of thumb, matches supported by multiple reference sequences expected to be more robust. However, due to incompleteness of the reference database, many species might have only one or few reference sequences available.


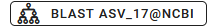
For additional diagnostics of the sequence match specificity, it is highly recommended to verify the match by checking the phylogenetic tree of the wider range of related taxa from NBCI Genbank. This can be done directly from the Pest Alert Tool results in a list view mode, by pressing the ‘BLAST’ button next to the species of interest, i.e. .

In the example below we select *Pseudopolydora paucibranchiata* species for phylogenetic similarity investigation. By pressing the button, the sequence from the user’s dataset is submitted to NCBI’s phylogenetic tree generation engine which runs BLAST pairwise alignments. When the tree is ready it is indicated by green button ‘VIEW TREE’. This link on the button will take the user to the Tree View on NBCI platform. For more details about interacting with the Tree View on NCBI look at the NCBI tutorial (<https://www.ncbi.nlm.nih.gov/tools/gbench/tutorial19/>). To verify the match, users should check the location of the highlighted query (the submitted sequence). A robust match is expected to be on a branch with the same species and be separated from other species (also verify that the gene matches the submitted sequencing input i.e. COI or 18S). Note, that on small screens the tree might be presented with collapsed branches. To zoom in, right-click on the branch and select “Expand All” option.


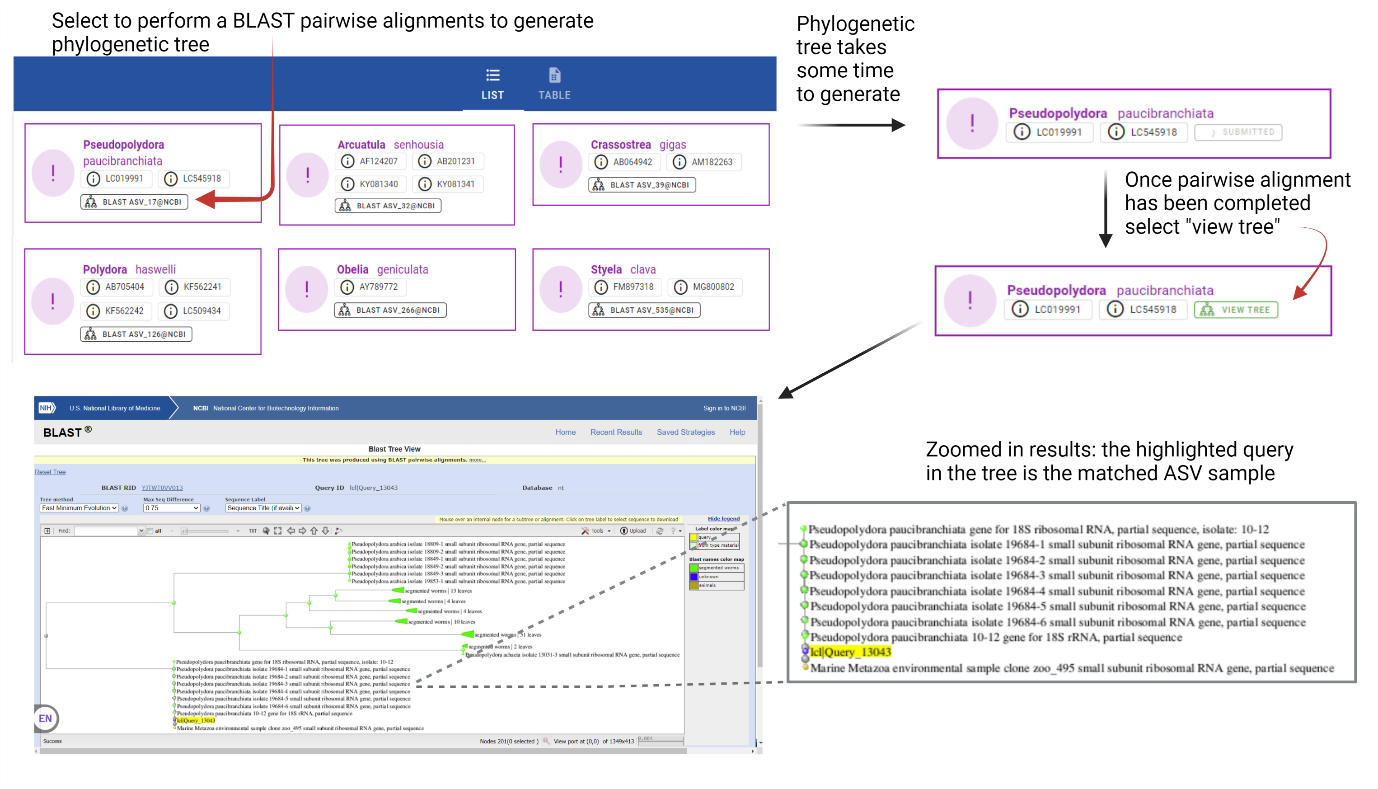


**Figure 8:** Performing a phylogenetic tree using BLAST pairwise alignment to verify detection of a putative pest
