# Supplementary material for: Pest Alert Tool—a web-based application for flagging species of concern in metabarcoding datasets
Source: Nucleic Acids Res. 2023 May 19;51(W1):W438–42. doi: 10.1093/nar/gkad364 (PMC10320087; doi:10.1093/nar/gkad364)
Supplement: gkad364_Supplemental_Files [file gkad364_supplemental_files.zip › Supp2_Zaiko_etal_PAT_FASTA_gen.docx]

# Supplementary 2: A guide for processing raw sequencing files

Please be aware that the following pre-processing pipeline for raw sequences is just one example and closely follows the DADA^2^ tutorial (<https://benjjneb.github.io/dada2/>).

Text highlighted in red should be edited to indicate actual file location on the drive.

# Loading of required libraries in R and creating of environments.

Cutadapt needs to be installed on your local machine and can be downloaded from miniconda (<https://docs.conda.io/en/latest/miniconda.html>). Then define the path in your R script as below.

library(dada2); packageVersion("dada2")

library(data.table)

library(openssl)

library(theseus)

library(tidyverse)

library(Biostrings)

library(ggpubr)

cutadapt = "*provide path to your cutadapt environment*"

path_main <- getwd()

path_fastq <- paste0(path_main)

path_results <- paste0(path_main,"/*folder_name*")

if(!dir.exists(path_results)) dir.create(path_results)

forward_primer=c("*your forward primer sequence*"); reverse_primer=c("*your reverse primer sequence*")

tL = c(*225,216*) # Truncation length for the forward and reverse reads, needs to be adapted to your amplicon size.

overlap = "--overlap 17" # Example minimum overlap for primer matching with cutadapt, needs to be adjusted to your amplicons

# Step 1

# Demultiplexing and primer removal

# Forward and reverse fastq filenames have format: SAMPLENAME_R1_001.fastq and SAMPLENAME_R2_001.fastq

fas_Fs_raw <- sort(list.files(path_fastq, pattern="R1_001.fastq.gz", full.names = TRUE))

fas_Rs_raw <- sort(list.files(path_fastq, pattern="R2_001.fastq.gz", full.names = TRUE))

fas_Fs_raw[2]

fas_Rs_raw[2]

FWD <- forward_primer

REV <- reverse_primer

FWD_RC <- dada2:::rc(FWD)

REV_RC <- dada2:::rc(REV)

path_cut <- file.path(path_results, "cutadapt")

if(!dir.exists(path_cut)) dir.create(path_cut)

fas_Fs_cut <- file.path(path_cut, basename(fas_Fs_raw))

fas_Rs_cut <- file.path(path_cut, basename(fas_Rs_raw))

R1_flags <- paste(paste("-g", FWD, collapse = " "), paste("-a", REV_RC, collapse = " "))

R2_flags <- paste(paste("-G", REV, collapse = " "), paste("-A", FWD_RC, collapse = " "))

for(i in seq_along(fas_Fs_raw)) {

cat("Processing", "-----------", i, "/", length(fas_Fs_raw), "-----------\n")

system2(cutadapt, args = c(R1_flags, R2_flags,

"--discard-untrimmed",

"--max-n 0",

overlap,

# Optional strong constraint on expected length

#paste0("-m ", 250-nchar(FWD)[1], ":", 250-nchar(REV)[1]),

#paste0("-M ", 250-nchar(FWD)[1], ":", 250-nchar(REV)[1]),

"-o", fas_Fs_cut[i], "-p", fas_Rs_cut[i],

fas_Fs_raw[i], fas_Rs_raw[i]))

}

out_1 <- ShortRead::qa(fas_Fs_raw)[["readCounts"]][,"read", drop = FALSE]

head(out_1)

# Inspect read quality profiles

pF <- plotQualityProfile(sample(fas_Fs_cut, replace = FALSE, size = ifelse(length(fas_Fs_cut) < 100, length(fas_Fs_cut), 100)),aggregate = TRUE) + ggplot2::labs(title = "Forward")

pR <- plotQualityProfile(sample(fas_Rs_cut, replace = FALSE, size = ifelse(length(fas_Rs_cut) < 100, length(fas_Rs_cut), 100)),aggregate = TRUE)+ ggplot2::labs(title = "Reverse")

test = ggarrange(pF,pR, nrow = 2)

ggsave(filename = file.path(path_results, "Read_quality_profile_aggregated.pdf"), plot = test, width = 6, height = 8)

# Step 2: Quality filtering

# Place filtered files in filtered/ subdirectory

cat("\nPerforming quality filtering\n")

path_process <- path_cut

fnFs <- sort(list.files(path_process, pattern="R1_001.fastq.gz", full.names = TRUE))

fnRs <- sort(list.files(path_process, pattern="R2_001.fastq.gz", full.names = TRUE))

#Extract sample names, assuming filenames have format: SAMPLENAME_XXX.fastq

sample.names <- sapply(strsplit(basename(fnFs), "_S\\d+"), `[`, 1)

filtFs <- file.path(path_results, "filtered", paste0(sample.names, "_F_filt.fastq.gz"))

filtRs <- file.path(path_results, "filtered", paste0(sample.names, "_R_filt.fastq.gz"))

out_2 <- filterAndTrim(fnFs, filtFs, fnRs, filtRs, trimLeft=c(0,0),

truncLen=tL,maxN=0, maxEE=c(2,2), truncQ=2,

rm.phix=TRUE,compress=TRUE, multithread=cores) # On Windows set multithread=FALSE, maxEE parameter needs to be checked based on quality.

head(out_2,20)

# Step 3: Learn error rates

cat("\nLearning error rates\n")

filtFs <- paste0(file.path(path_results, "filtered", list.files(path=paste0(path_results,"/filtered"), pattern="_F_filt.fastq.gz")))

filtRs <- paste0(file.path(path_results, "filtered", list.files(path=paste0(path_results,"/filtered"), pattern="_R_filt.fastq.gz")))

errF <- learnErrors(filtFs, multithread=cores)

errR <- learnErrors(filtRs, multithread=cores)

perrF <- plotErrors(errF, nominalQ = TRUE) + ggplot2::labs(title = "Error Forward")

perrR <- plotErrors(errR, nominalQ = TRUE) + ggplot2::labs(title = "Error Reverse")

fig = ggarrange(perrF,perrR, nrow = 2)

ggsave(filename = paste0(path_results,"/Error_rates_learning.pdf"), fig ,width = 6, height = 8)

# Step 4: Dereplication

exists <- file.exists(filtFs)

derepFs <- derepFastq(filtFs[exists], verbose=TRUE)

derepRs <- derepFastq(filtRs[exists], verbose=TRUE)

# Name the derep-class objects by the sample names

names(derepFs) <- sapply(strsplit(basename(filtFs), "_F_filt"), `[`, 1)

names(derepRs) <- sapply(strsplit(basename(filtFs), "_F_filt"), `[`, 1)

# Step 5: Sample inference

cat("\nDenoising the data\n")

dadaFs <- dada(derepFs, err=errF, multithread=cores)

dadaRs <- dada(derepRs, err=errR, multithread=cores)

# Step 6: Merging forward and reverse reads

cat("\nMerging reads\n")

mergers <- mergePairs(dadaFs, derepFs, dadaRs, derepRs, minOverlap = 10, maxMismatch = 0, verbose=TRUE)

# Step 7: Construct feature table

seqtab <- makeSequenceTable(mergers)

dim(seqtab)

# Inspect distribution of sequence lengths

table(nchar(getSequences(seqtab)))

# Step 8: Remove chimeras

cat("\nRemoving chimeras\n")

seqtab.nochim <- removeBimeraDenovo(seqtab, method="consensus", multithread=cores, verbose=TRUE)

dim(seqtab.nochim)

saveRDS(seqtab.nochim, file = paste0(path_results,"/seqtab.nochim.rds"))

uniquesToFasta(getUniques(seqtab.nochim), fout= paste0(path_results,"/uniqueSeqs.fasta"),ids=as.character(as.list(md5(names(getUniques(seqtab.nochim))))))

#Step 9:

Now use the saved “uniqueSeqs.fasta” file as input and drag it directly into the Pest Alert Tool interface for screening of marine non-indigenous species (NIS) as well as unwanted and notifiable marine organisms in New Zealand.
