# Supplementary material for: Pest Alert Tool—a web-based application for flagging species of concern in metabarcoding datasets
Source: Nucleic Acids Res. 2023 May 19;51(W1):W438–42. doi: 10.1093/nar/gkad364 (PMC10320087; doi:10.1093/nar/gkad364)
Supplement: gkad364_Supplemental_Files [file gkad364_supplemental_files.zip › Supp3_Zaiko_etal_PAT_Database_gen.docx]

# Supplementary 3: A guide for creating a customized reference database for a user-defined list of species of concern.

**Dependencies:**

The downloading of sequences for the creation of the database is undertaken using CRABS {Jeunen, 2022 #1654}. Software instructions can be found here: <https://github.com/gjeunen/reference_database_creator>.

Download the accession2taxid, nodes.dmp and names.dmp from NCBI.

Have BLAST+ installed. https://blast.ncbi.nlm.nih.gov/doc/blast-help/downloadblastdata.html#downloadblastdata

**Downloading reference files:**

CRABS can be used to download reference sequences from several online repositories (NCBI, BOLD, EMBL or MitoFish). As PAT is designed for use with the 18S rRNA and COI genes we have utilized NCBI and BOLD and examples for both are below.

Example for downloading NCBI reference files:

crabs db_download \

--source ncbi \

--database nucleotide \

--query 'Acantholobulus[ORGN] OR Acanthophora[ORGN] AND (co1[gene] OR cox1[gene] OR coi[gene] OR mtco1[gene]) AND (350[SLEN] : 50000[SLEN])' \

--output ncbi.coi.fasta \

--keep_original no \

--email [user@email.com](mailto:user@email.com) \

--batchsize 10000

In the example two genera containing non-indigenous species are queried for the COI gene using a variety of different naming options for the gene and with length limits of 350-50000bp. For the 18S gene an example --query command would be:

--query 'Acantholobulus[ORGN] OR Acanthophora[ORGN] AND (18S[All Fields]) AND (350[SLEN] : 50000[SLEN])'

The query list may have to be shortened if too many genera are searched for, and multiple output files created. These can then be merged in later steps.

Example for downloading BOLD reference files:

crabs db_download \

--source bold \

--database 'Acantholobulus|Acanthophora' \

--output bold.coi.fasta

**Merge into one database per gene:**

If two outputs have had to be produced for a database or if you want to combine the results from two databases into one the db_merge function can be used. In the below example 2 fasta files were obtained from the NCBI and BOLD database and then combined.

crabs db_merge \

--output COI.combined.fasta \

--uniq yes \

--input ncbi.coi_v1.fasta ncbi.coi_v2.fasta bold.coi_v1.fasta bold.coi_v2.fasta

The --uniq tag allows only unique accession numbers to be kept.

Clean up any N’s in the sequence that occur at the beginning or end of the sequence.

awk '{if (/>.*/) {print} else { sub(/^N*/, "")sub(/N*$/, ""); print}}' COI.combined.fasta > COI.combined_cleaned.fasta

**Assign taxonomy to the reference sequences:**

Assign taxonomy to the reference sequences based on the NCBI taxonomy.

crabs assign_tax \

--input COI.combined_cleaned.fasta \

--output COI.combined.tax.tsv \

--acc2tax referencedb/nucl_gb.accession2taxid \

--taxid referencedb/nodes.dmp --name referencedb/names.dmp

**Clean-up the database:**

crabs seq_cleanup --input COI.combined.tax.tsv --output COI.combined.tax.derep.cleaned.tsv --minlen 350 --maxlen 50000 --maxns 1 --enviro no --species yes --nans 0

Don’t include any environmental sequences or those with missing taxonomic information. Only allow sequences with 1 or fewer ambiguous bases.

**Output the reference sequences in fasta format and clean up the headers:**

crabs tax_format --input COI.combined.tax.derep.cleaned.tsv --output NIS.COI.db.fasta --format dads

awk 'BEGIN{ FS=OFS=" " }{ gsub("\:.*", "", $1) }1' NIS.COI.db.fasta > NIS.COI.db.1.fasta

awk '/^>/ {print $1 ";", $3; next}1' NIS.COI.db.1.fasta > NIS.COI.db.2.fasta

awk '/^>/ {gsub(/; /, ";")}; 1' NIS.COI.db.2.fasta > NIS.COI.db.final.fasta

Note that fasta headers have to be <50 characters long to be made into a BLAST database so some may need to be trimmed.

**Make the blast database:**

makeblastdb -in NIS.COI.db.final.fasta -parse_seqids -blastdb_version 5 -dbtype nucl

If the user wants to use their own database this can be added to a local installation of the tool in the /backend/db source code.

The file name for the database should be changed to either **NIS.rRNA18S.db.final.fasta** or **NIS.COI.db.final.fasta**. In the /db/routes.py file the NIS should be updated to the user defined list.
